# Vascular Heterogeneity With a Special Focus on the Hepatic Microenvironment

**DOI:** 10.3389/fphys.2020.591901

**Published:** 2020-11-11

**Authors:** Johannes Robert Fleischer, Chiara Angelina Jodszuweit, Michael Ghadimi, Tiago De Oliveira, Lena-Christin Conradi

**Affiliations:** Department of General, Visceral and Pediatric Surgery, University Medical Center Göttingen, Göttingen, Germany

**Keywords:** vasculature, heterogeneity, microenvironment, liver metastases, angiogenesis, vessel co-option

## Abstract

Utilizing single-cell sequencing, recent studies were able to analyze at a greater resolution the heterogeneity of the vasculature and its complex composition in different tissues. Differing subpopulations have been detected, distinguishable only by their transcriptome. Designed to provide further insight into the heterogeneity of the functional vascular tissue, endothelial cells have been the main target of those studies. This review aims to present a synopsis of the variability of the different vascular beds, their endothelial variety, and the supporting cells that allow the vessels to serve their various purposes. Firstly, we are going to chart vascular tissue heterogeneity on a cellular level, describing endothelial diversity as well as stromal microenvironmental variety and interaction in a physiological setting. Secondly, we will summarize the current knowledge of pathological vessel formation in the context of cancer. Conventional anti-tumor therapeutic targets as well as anti-angiogenetic therapy is frequently limited by poor response of the tumor tissue. Reasons for moderate response and resistance to treatment can be found through different drivers of angiogenesis, different mechanisms of blood supply, but also in poorly understood tissue diversity. Based on this, we are comparing how pathologies alter the normal structure of vascular tissues highlighting the involved mechanisms. Lastly, illustrating the concept above, we will focus on the hepatic microenvironment, an organ of frequent metastatic spreading (e.g., from colorectal, breast, and lung cancers). We will address how the hepatic vasculature usually develops and subsequently we will describe how common liver metastases vary in their vasculature and the way they supply themselves (e.g., angiogenesis versus vessel co-option).

## Physiological Vessel Heterogeneity

Heterogeneity of the vasculature can be determined and discussed on different levels within the vascular tree and with respect to multi-omics analyses. This review first wants to recapitulate microscopic features of the body’s vessels as a brief introduction to the topic of vascular heterogeneity by connecting it to familiar knowledge. Secondly, we want to focus on the transcriptome level of heterogeneity, as we understand single-cell RNA sequencing (scRNAseq) as the current state-of-the-art technique in high-throughput analyses.

### Basic Composition of the Vasculature

**FIGURE 1:**
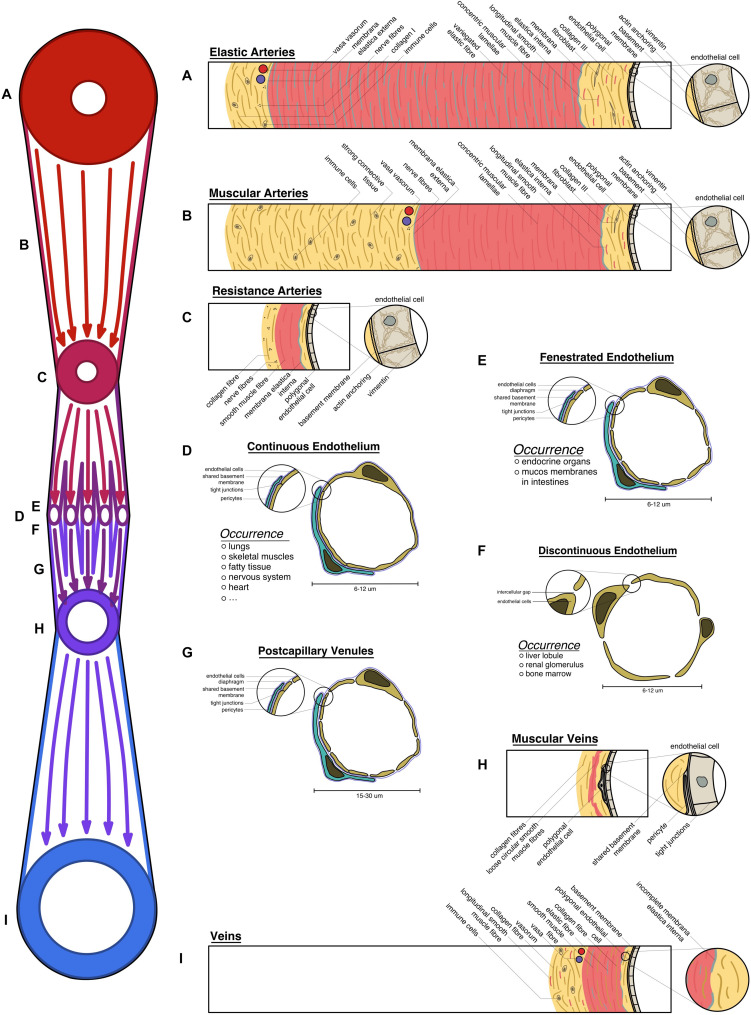
Overview of the vascular system: schematic transversal depiction of the structural elements of all vessels under homeostatic conditions: **(A)** elastic arteries, expressing strong muscular lamellae with variegated elastic fibers; **(B)** muscular arteries with strong connective tissue; **(C)** resistance arteries; **(D)** continuous endothelium; **(E)** fenestrated endothelium; **(F)** discontinuous endothelium; **(G)** postcapillary venules; **(H)** muscular veins; **(I)** large veins exhibiting longitudinal muscle fibers in their connective tissue. **(A–I)** All vessel types are laid on the schematic vascular tree (on the left), according to their physiological position.

All blood vessels can be characterized according to their function in transporting blood and nutrients (Pawlina, 2020). The largest arteries, in closest proximity to the heart, experience the highest pressure gradient and are tasked in transforming those pressure peaks into an even flow (Drake et al., 2015). These elastic arteries exhibit polygonal endothelial cells that are aligned in the direction of flow, and are reinforced with a strong cytoskeleton and actin filaments anchoring the cells to the basement membrane to cope the shear stress. To withstand and level out pressure peaks, elastic arteries are equipped with strong concentric muscular lamellae variegated with elastic fibers, assuming high volume compliance to store up to half of the cardiac output and discharge it during low-pressure states (see Figure 1A; Boron and Boulpaep, 2003; Pape et al., 2014; Welsch et al., 2014).

These arteries turn into their muscular counterparts, which are less elastic with the primary focus on withstanding the blood pressure. Consequently, the intima of those vessels closely resembles the previously described phenotype, however, the media is lacking elastic fibers. The connective tissue, holding the arteries in place is strongly developed (see Figure 1B; Welsch et al., 2014; Drake et al., 2015).

In the microcirculation small arterioles regulate the perfusion of the vascular bed. Ordinarily, constriction of the thin smooth muscle lamellae allows only 25% of the capillary bed to be perfused (Boron and Boulpaep, 2003; Drake et al., 2015). Creating the main body of vascular resistance leads to their name of resistance arteries. This interaction is tightly coordinated, hence a dense neural plexus can be found in their adventitia (see Figure 1C; Welsch et al., 2014; Pawlina, 2020). These vessel subtypes display a prominent membrana elastica interna that can be utilized to distinguish arteries from veins by using histochemistry stainings, such as Verhoeff’s elastica stain or more specifically resorcin-fuchsin (Puchtler and Waldrop, 1979).

Capillaries are ubiquitously found in the body, given that oxygen can only traverse 2 mm through tissue (Griffith et al., 2005). Needing to fulfill every tissue specific demand, more structural heterogeneity is observed in all parts of the human body. Tissues like the brain, heart, and skeletal muscle have little physiological need to exchange big particles. However, some sensitive organs have a high need for protection against harmful substances (e.g., the blood–brain-barrier, blood–testis-barrier). Thus, a continuous endothelium with varying levels of occluding junctions is present in those areas. Endocrine organs, bowel mucosa, or adipose tissue have a higher need for facilitated diffusion, which is met by capillaries with fenestrated endothelium and diaphragms closing the fenestrations. Liver lobules, the bone marrow, or the glomeruli of the kidneys have the highest need for vessel permeability, thus displaying a discontinuous endothelium with intercellular gaps (see Figures 1D–F; Welsch et al., 2014; Pawlina, 2020).

Recollecting and supplying the blood back to the heart, venules are bigger capillaries at first, with fenestrations, allowing immune cells to trespass into the surrounding tissue (diapedesis) (see Figure 1G). In secondary lymphoid tissue, high endothelial venules can be found that facilitate recirculation of naive lymphocytes through lymphoid organs (see Figure 1G; Veerman et al., 2019).

Closer to the heart, those venules start to exhibit loose circular smooth muscle fibers and strong connective tissue, embedding the vessel into its environment (see Figure 1H; Welsch et al., 2014).

Big veins almost reassemble the structure of the arteries that they are running with. Contrarily, they have a bigger lumen compared to arteries and store up to 80% of the total blood volume. They have in general thinner structures, as they do not have to withstand the high pressure levels present in the arterial system. However, as veins can experience negative pressure, longitudinal smooth muscle fibers can be found in the adventitia, allowing them to keep the lumen open during low-pressure states (see Figure 1I). Depending on the location those structures vary as venules and veins embedded into tight connective tissue, like the dura mater, do not show any musculature as they are not in need to regulate their wall pressure. Veins located in the lower extremities often exhibit venous valves to inhibit backflow (Welsch et al., 2014; Pawlina, 2020).

### Latest Insights Into Vessel Heterogeneity

#### Endothelial Cell Heterogeneity

Endothelial cells (ECs) are the innermost lining of all blood vessels. Approximations put the length of all vessels combined above 100.000 km. Dysfunctional ECs are a predictor for cardiovascular disease (Widlansky et al., 2003) but are also a relevant contributor component to other diseases, as observed in newly formed aberrant cancer vessels. Therefore, a profound understanding of their pathophysiology is necessary to provide patients with better therapies. It is well established that ECs are heterogeneous and vary between different tissue types, within the vascular tree of an organ and even between neighboring (Aird, 2012), yet an exhaustive and in-depth understanding is still not established. With the emerge of single-cell sequencing techniques in the last years, the resolution in which we can detect heterogeneity has been increased noticeably (Sabbagh et al., 2018; Vanlandewijck et al., 2018; Lukowski et al., 2019; Goveia et al., 2020; Jambusaria et al., 2020; Kalucka et al., 2020; Rohlenova et al., 2020). We are going to review and consider recent findings on EC diversity, and compare them to the available knowledge.

##### General EC makers

Although ECs are heterogeneous, they can be identified in a mixture of cell types by sorting for conserved features that all ECs express. The platelet-endothelial cell adhesion molecule 1 (*Pecam1*) also known as cluster of differentiation 31 (*CD31*) can be used to distinguish ECs. As *Pecam1/CD31* co-stains immune cells, additional sorting for *CD34*, von Willebrand factor (*vWF*), or vascular E-Cadherin (VE-cadherin) has to be done to securely identify ECs (Ghilardi et al., 2008; Cleuren et al., 2019). Another study has used an expression pattern of *Pecam1, Cdh5*, and *Tie1* to identify ECs (Feng et al., 2019; see Table 1).

**TABLE 1:** General EC marker genes.

Marker	Remarks	Associated function	Species	Study
**General EC marker genes**				
*Pecam1*	Co-stains immune cells	Adhesion molecule	mouse	Ghilardi et al., 2008; Cleuren et al., 2019; Feng et al., 2019
*CD34*		Adhesion molecule	mouse	Ghilardi et al., 2008; Cleuren et al., 2019
*vWF*	Mostly expressed in cells from large vessels	Glycoprotein involved in hemostasis	mouse	Ghilardi et al., 2008; Cleuren et al., 2019
*VE-cadherin*		Adherends junctions	mouse	Ghilardi et al., 2008; Cleuren et al., 2019; Feng et al., 2019
*Tie1*		Angiopoietin receptor	mouse	Feng et al., 2019

##### Inter-tissue heterogeneity

As cells that line the inner surface of the body’s blood vessels, ECs are the key regulatory cells in the crosstalk between tissue and the overall systemic circulation. They face systemic challenges such as regulation of the blood pressure, mediation of the immune response, or initiation of hemostasis, but also tasks such as facilitated diffusion or cellular barrier functions. Consecutively, it seems only natural that ECs, which have to meet such heterogeneous tasks, differ on all levels of multi-omics. Several recent studies have contributed to a further understanding of how heterogeneous ECs from different organs are (Sabbagh et al., 2018; Cleuren et al., 2019; Feng et al., 2019; Goveia et al., 2020; Kalucka et al., 2020). The following paragraph reports on these findings. However, the origin of ECs (wild type mice, transgenic mice, *ex vivo*), the methods of how the single-cell level was achieved (enzymatic, mechanical, or both) and the general study designs differ. As proven by Cleuren et al. (2019), those factors significantly impact the transcriptome, hence differences in the experimental methodology limit the comparability and implementation between these studies.

ECs in the brain, eye and testis are part of the blood–brain barrier, blood-retinal barrier or blood–testis barrier and express the highest degree of occlusion in continuous endothelium that can be found in the vascular system (Daneman and Prat, 2015; Mruk and Cheng, 2015; Díaz-Coránguez et al., 2017). Using scRNAseq on murine tissue samples, Kalucka et al. (2020) were able to verify previous reports (Su et al., 2011; Sweeney et al., 2019) that those ECs express differentially overexpressed gene sets involved in transmembrane transport. When performing differential analysis on 11 different murine organs, they identified *Pglyrp1* and *Lcn2* (related to the innate immune response) as novel marker genes that where solely expressed in brain and testis ECs, respectively (Kalucka et al., 2020). Feng et al. (2019) found *Slc2a1* and *Itm2a* to be uniquely expressed by brain ECs (Feng et al., 2019), while Cleuren et al. (2019) additionally described the genes *Rad54b, Zic3*, and *Slco1c1* as distinctive brain EC markers (Cleuren et al., 2019). Additionally, another single-cell study comparing ECs from four different murine tissues found that brain ECs upregulates the expression of genes encoding the membrane transporters *Mfsd2a, Slc2a1*, and *Slco1c1* (Sabbagh et al., 2018).

Transcriptomes of ECs from skeletal muscle tissue and the heart showed high expression of gene sets that were involved in membrane transport and redox homeostasis fitting to the abundance of oxygen and their resulting highly oxidative environment (Kalucka et al., 2020). In skeletal muscle, *Ssh2*, and *Nrp1* were found highly expressed (Kalucka et al., 2020).

In the heart, ECs not only line the coronary vessels, but also form the endocardium and the adjacent part of the ascending aorta. Here, Feng et al. (2019) show a high expression of the fatty acid transporting genes *Fabp4* and *Cd36* in the coronaries, *Npr3* (atrial natriuretic peptide receptor), and *Cytl1* in the endocardial ECs, and *Ehd3* and *Fam167b* in the aortic ECs (Feng et al., 2019). Also, *A2m* and *Itgbl1* have been described as endocardial EC marker genes (Cleuren et al., 2019). In synopsis with other sequencing data, varying levels of *Ehd3* expression in the aortic EC clusters are observed (Feng et al., 2019; Lukowski et al., 2019). Still, the origin of this variance remains to be determined, but as previously mentioned, differences in the techniques utilized in the workflow/analysis (e.g., underlying confounders) should be taken into consideration.

When observing the gene signature of lung ECs, MHC II genes were highly differentially expressed, suggesting their role in immune surveillance. This finding aligns with results from another recent single-cell study (Goveia et al., 2020). *Tmem100*, a transmembrane protein responsible for developmental endothelial differentiation and vascular morphogenesis and regulation of nociception, was identified as a marker gene to be exclusively expressed in lung tissue (Kalucka et al., 2020). Additionally, Cleuren et al. (2019) identified the immune system-related genes *Trgj1* and *Trim29* to be highly expressed in lung ECs. An interesting finding, reported by Feng et al. (2019), is the apparent heterogeneity between lung ECs, which can be traced back to the sex of the mouse that the ECs were harvested from. This gender difference was found in several organs, while others, such as brain ECs did not show this behavior.

Interestingly, comparing ECs from four different murine tissues, Sabbagh et al. (2018) found that liver ECs express genes that encode for scavenging receptors like *Fcgr2b, Stab2*, and *Clec4g* in line with the specializations of the tissue in question. *Dnase113* was found to be another liver EC marker, whereas *Clec4g* was confirmed by another independent group (Feng et al., 2019). Nevertheless, *Tmem132e, Ush1g*, and *Wnt9b* were described by Cleuren et al. (2019) as specific hepatic markers.

Karaiskos et al. (2018) focused on characterizing the heterogeneity within the murine renal glomerulus. They have established the existence of several subclusters within the glomerular ECs. The authors further detected an upregulation of *Ehd3*, which was suggested as a gene-specific marker to glomerular ECs by previous studies (George et al., 2011; Karaiskos et al., 2018). Summarizing, the relevant data presented by those studies (Feng et al., 2019; Lukowski et al., 2019) has to be taken into account in the execution and analysis of future scientific approaches. Nevertheless, *Ehd3* can be used as a glomerular EC marker gene within kidney cells. Other subpopulations showed the expression of *Jag1*, connected to EC pericyte (PC) crosstalk, as well as *Fbln5, Cxcl1*, and *Cldn5*, related to the regulation of angiogenesis, endothelial activation, and response to complement activation, respectively (Karaiskos et al., 2018). Other groups have also established *Gata5, Krtdap*, and *Lgr5* as genes upregulated in renal ECs (Cleuren et al., 2019).

Kalucka et al. (2020) conducted hierarchical clustering, a technique that establishes a pyramidal scheme that allows to examine relations between the different cell clusters. Including all identified subclusters, the authors found the tissue of origin accounting for most of the heterogeneity between the subtypes rather than the affiliation of different parts of the vascular tree. These findings indicate that capillary ECs are very adaptive to their environment expressing tissue-specific markers rather than generally conserved markers. This presumption is supported by another independent study (Cleuren et al., 2019).

While reporting on the heterogeneity of ECs within different tissues and vascular beds it is especially important to emphasize the finding that arterial and venous ECs of different tissues express congruent markers between 80 and 100% of all examined tissues (Kalucka et al., 2020). This finding implies a conservational phenomenon in these areas of the vascular tree. As capillaries express more heterogeneous markers, it seems that those vessels are more adaptive to their tissue environment (Kalucka et al., 2020).

**FIGURE 2:**
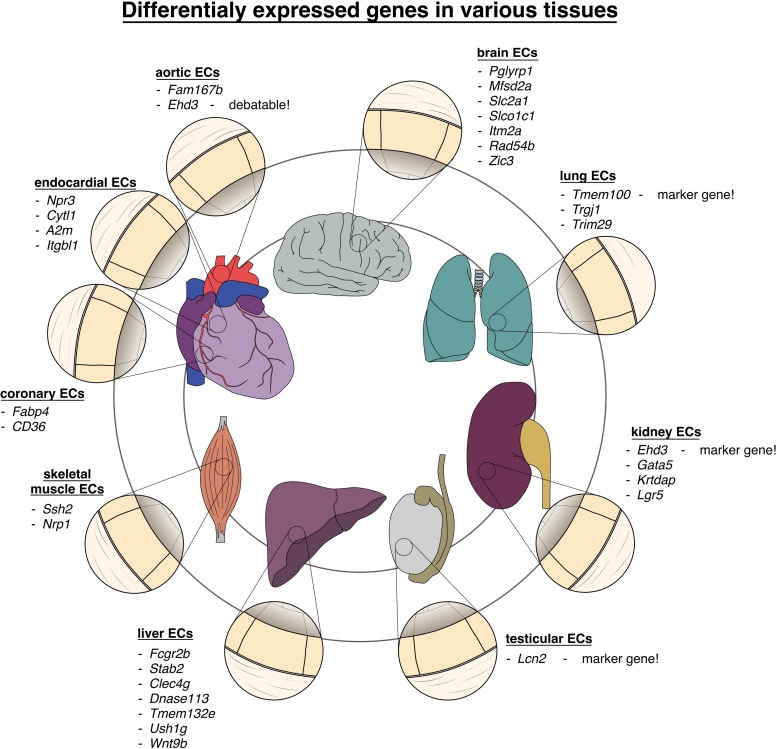
Vascular heterogeneity between different organs and tissues: chart of genes differentially upregulated in endothelial cells in various tissues of the body.

For a comprehensive listing of all marker genes named see Table 2 and Figure 2.

**TABLE 2:** Differentially expressed genes in different tissues.

Organ	Marker	Remarks	Associated function	Species	Study
**Inter-tissue heterogeneity**					
Testis	*Lcn2*	Marker gene	Innate immune response	mouse	Kalucka et al., 2020
Brain	*Pglyrp1*	Marker gene	Innate immune response	mouse	Kalucka et al., 2020
Brain	*Mfsd2a*		Transporter	mouse	Sabbagh et al., 2018
Brain	*Slc2a1*		Transporter	mouse	Sabbagh et al., 2018; Feng et al., 2019
Brain	*Slco1c1*		Transporter	mouse	Sabbagh et al., 2018; Cleuren et al., 2019
Brain	*Itm2a*		Integral membrane protein/Immune activation	mouse	Feng et al., 2019
Brain	*Rad54b*		DEAD-like helicase superfamily	mouse	Cleuren et al., 2019
Brain	*Zic3*		Cerebellum ZIC family	mouse	Cleuren et al., 2019
Skeletal muscle	*Ssh2*		Protein phosphatase slingshot homolog 2	mouse	Kalucka et al., 2020
Skeletal muscle	*Nrp1*		Neuropilin 1, role in angiogenesis, cell survival migration, and invasion	mouse	Kalucka et al., 2020
Coronaries	*Fabp4*		Carrier protein fatty acids	mouse	Feng et al., 2019
Coronaries	*CD36*		Fatty acid translocase	mouse	Feng et al., 2019
Endocardial ECs	*Npr3*		Atrial natriuretic peptide receptor	mouse	Feng et al., 2019
Endocardial ECs	*Cytl1*		Cytokine-like 1	mouse	Feng et al., 2019
Endocardial ECs	*A2m*		Antiprotease	mouse	Cleuren et al., 2019
Endocardial ECs	*Itgbl1*		Integrin subunit beta like 1	mouse	Cleuren et al., 2019
Aortic ECs	*Fam167b*			mouse	Feng et al., 2019
Aortic ECs	*Ehd3*	Debatable!	Endocytic trafficking, moonlighting protein	mouse	Feng et al., 2019; Lukowski et al., 2019
Lung	*Tmem100*	Marker gene	Transmembrane protein 100	mouse	Kalucka et al., 2020
Lung	*Trgj1*		T-cell receptor joining 1	mouse	Cleuren et al., 2019
Lung	*Trim29*		TRIM protein family	mouse	Cleuren et al., 2019
Liver	*Fcgr2b*		Scavenging receptor	mouse	Sabbagh et al., 2018
Liver	*Stab2*		Scavenging receptor	mouse	Sabbagh et al., 2018
Liver	*Clec4g*		Scavenging receptor	mouse	Sabbagh et al., 2018; Feng et al., 2019
Liver	*Dnase113*			mouse	Feng et al., 2019
Liver	*Tmem132e*		Transmembrane protein	mouse	Cleuren et al., 2019
Liver	*Ush1g*			mouse	Cleuren et al., 2019
Liver	*Wnt9b*		Wnt Family Member 9B	mouse	Cleuren et al., 2019
Glomerular kidney	*Ehd3*	Marker gene	Endocytic trafficking, moonlighting protein	mouse	George et al., 2011; Karaiskos et al., 2018
Kidney	*Gata5*		Transcription factor	mouse	Cleuren et al., 2019
Kidney	*Krtdap*		Keratinocyte differentiation-associated protein	mouse	Cleuren et al., 2019
Kidney	*Lgr5*		Member Wnt signaling pathway	mouse	Cleuren et al., 2019

##### Intra-tissue heterogeneity/heterogeneity within the vascular tree

The fact that ECs of different parts of the vascular hierarchy are heterogeneous is also well established (Yamamoto et al., 1998; Gustavsson et al., 2010). For example, *Vcam-1* and *vWF* expression correlates to vessel size, being almost absent in small capillaries and most abundant in the big vessels (Yamamoto et al., 1998; Gustavsson et al., 2010). However, the exact relation of the different ECs was at the time of the discovery unclear. Using the sorting points in neighborhood method (SPIN; Tsafrir et al., 2005), a form of pseudo-time trajectory, Vanlandewijck et al. (2018) established a one-dimensional trajectory using ECs of murine brain. When analyzing the trajectory for previously described marker genes, they found the arterial markers *Bmx, Efnb2, Vegfc*, and *Sema3g* (Ekman et al., 1997; Wang et al., 1998; Hogan et al., 2009; Kutschera et al., 2011) to be expressed at one end, presumed to be the arterial end. The venous marker *Nr2f2* (Hirashima and Suda, 2006) peaked at the opposing end of the range, while the capillary marker *Mfsd2a* (Nguyen et al., 2014) was found to be expressed in the middle of the trajectory (Vanlandewijck et al., 2018). Therefore, the expression pattern of *Vcam-1* and *vWf* at the opposing sides of the range, but not the middle (denoted as the capillary region), fitted to the description that they are only expressed in arteries and veins. *Tfrc* and *Slc16a1* were found to be expressed on the middle left part of the trajectory, fitting to the previously described expression in capillaries and veins (Vanlandewijck et al., 2018). The gradual decline in expression of genes along with the projection proved that ECs are exhibiting a continuous phenotype, coined zonation (Vanlandewijck et al., 2018), rather than showing discreet phenotypes.

Other patterns of markedly expressed genes that can be found in arteries are *Ephrin B2, Alk1, Dll4, NRP1, Depp, Hey1* and *Hey2, EPAS1* while veins express a pattern of *Ephrin B2, Eph B4, NRP2*, and *COUP−TFII* (Cheng et al., 2002; Aird, 2007; Ribatti et al., 2020).

**FIGURE 3:**
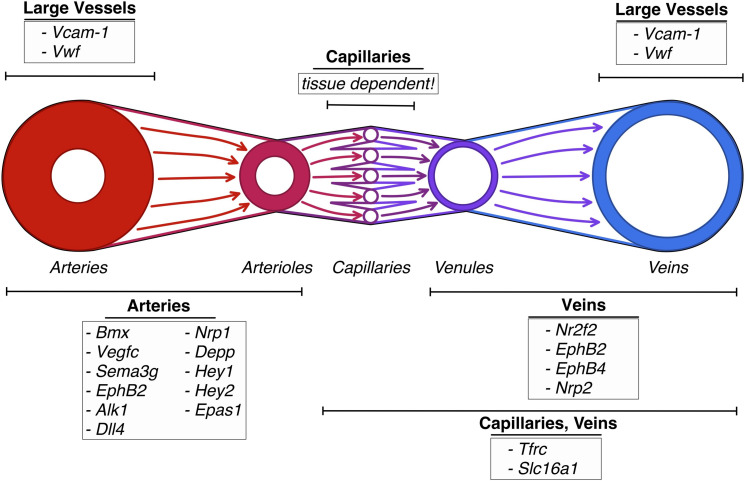
Vascular heterogeneity between sections of the vascular tree: schematic depiction of genes differentially upregulated in endothelial cells in the different sections of the vascular tree.

For a comprehensive listing of all named genes see Table 3 and Figure 3.

**TABLE 3:** Differentially expressed marker genes in the vascular tree.

Vascular tree	Marker	Name/function	Species	Study
**vascular tree heterogeneity**				
Large vessels	*Vcam-1*	Vascular cell adhesion molecule 1	Mouse	Yamamoto et al., 1998; Gustavsson et al., 2010
Large vessels	*Vwf*	von Willebrand factor	Mouse	Yamamoto et al., 1998; Gustavsson et al., 2010
Arteries	*Bmx*	Cytoplasmic tyrosine kinase	Mouse, human	Ekman et al., 1997
Arteries	*Efnb2*	Transmembrane protein (receptor)	Mouse	Wang et al., 1998
Arteries	*Vegfc*	Vascular endothelial growth factor C	Zebrafish	Hogan et al., 2009
Arteries	*Sema3g*	Endothelial cell-expressed class 3 semaphorin	Mouse	Kutschera et al., 2011
Arteries	*EphB2*	Ephrin type-B receptor 2	Mouse	Cheng et al., 2002; Aird, 2007; Ribatti et al., 2020
Arteries	*Alk1*	Cell-surface receptor	Mouse	Cheng et al., 2002; Aird, 2007; Ribatti et al., 2020
Arteries	*Dll4*	Notch ligand	Mouse	Cheng et al., 2002; Aird, 2007; Ribatti et al., 2020
Arteries	*Nrp1*	Neuropilin 1	Mouse	Cheng et al., 2002; Aird, 2007; Ribatti et al., 2020
Arteries	*Depp*	Decidual protein induced by progesterone	Mouse	Cheng et al., 2002; Aird, 2007; Ribatti et al., 2020
Arteries	*Hey1*	Transcription factor	Mouse	Cheng et al., 2002; Aird, 2007; Ribatti et al., 2020
Arteries	*Hey2*	Transcription factor	Mouse	Cheng et al., 2002; Aird, 2007; Ribatti et al., 2020
Arteries	*Epas1*	Endothelial PAS domain-containing protein 1	Mouse	Cheng et al., 2002; Aird, 2007; Ribatti et al., 2020
Capillaries, veins	*Tfrc*	Transferrin receptor	Mouse	Vanlandewijck et al., 2018
Capillaries, veins	*Slc16a1*	Solute carrier 16a1	Mouse	Vanlandewijck et al., 2018
Veins	*Nr2f2*	COUP-TFII	Mouse, human, zebrafish	Hirashima and Suda, 2006
Veins	*EphB2*	Ephrin type-B receptor 2	Mouse	Cheng et al., 2002; Aird, 2007; Ribatti et al., 2020
Veins	*EphB4*	Ephrin type-B receptor 4	Mouse	Cheng et al., 2002; Aird, 2007; Ribatti et al., 2020
Veins	*Nrp2*	Neuropilin 2	Mouse	Cheng et al., 2002; Aird, 2007; Ribatti et al., 2020

It was also found that transcription factors such as *Hey2, Junb*, or *Nr4a2* which are involved in arterial endothelial cell differentiation, regulation of cell proliferation and response to hypoxia respectively, were significantly expressed in the arterial ECs, while transporter genes dominated in capillaries and veins, suggesting that the prominent trans-endothelial transport is increased in those regions (Vanlandewijck et al., 2018).

Recently, the heterogeneity within bone marrow endothelial cells (BMEC) has been evaluated (Baryawno et al., 2019). BMECs expressed the established markers *Pecam1, Cdh5, Kdr*, and *Emcn* (Rafii et al., 2016) and are related to each other on a continuous trajectory. Sinusoidal BMECs show a pattern of high *Flt4* expression (encoding for VEGFR3) and low *Ly6a* expression (SCA-1). The cluster of arteriolar BMECs could be distinguished from the sinusoidal BMEC by an inverted pattern of low *Flt4* expression and high *Ly6a* expression (Baryawno et al., 2019). Those findings align with previously published reports (Hooper et al., 2009; Itkin et al., 2016). The third cluster that was identified was understood to be a subcluster of the arteriolar BMECs. Those ECs showed an exclusive but heterogeneous expression of *vWF*. Furthermore, CD34 which has been described as an associated marker for arteriolar BMECs, was found in the third identified cluster (Coutu et al., 2017), interestingly lacking the expression of the sinusoidal marker *Il6st* (Baryawno et al., 2019).

##### Intra-tissue heterogeneity/cell-to-cell variation

Recently, Lukowski et al. (2019) paid special attention to cell-to-cell heterogeneity in EC populations within the murine aorta, a phenomenon already described (Eichmann et al., 2005; Adams and Alitalo, 2007). Previously, Patel et al. (2017) described the existence of endovascular progenitor cells (EVP) within the vascular beds. Those cells are in contrast to definitive differentiated ECs which represent a successive cell type. Later, it was confirmed that all ECs where related to each other on a seamless trajectory rather than expressing a discreet and isolated phenotype (Patel et al., 2017; Donovan et al., 2019). EVPs express a stringent surface marker profile *CD34^+^ CD45^–^ CD31^*lo*^ VEGFR2*^–^ and increased expression of *VEGFR2* and *CD31* when transitioning to definitive differentiated ECs (Lukowski et al., 2019). Through differential analysis, definitive differentiated ECs could be characterized by an increase in *Pecam1* and *Cdh5* expression, while EVPs expressed high levels of *Pdgfra, Il33*, and *Sox9* (Lukowski et al., 2019).

#### Heterogeneity in Pericytes

In an effort to study heterogeneity within PCs, Vanlandewijck et al. (2018) identified PCs among other cell types within murine brain samples by applying known PC gene patterns to their data set. Sorting for the presence of canonical PC markers such as *Pdgfrb, Cspg4*, and *Des* and excluding cells expressing smooth muscle cell (SMC) markers like *Acta2* and *TagIn*, as well as fibroblast markers *Pdgfra, Lum*, and *Dcn*, the authors established a likely PC population within their murine brain sample. Moreover, they noted the shortcomings of this procedure which are that *Acta2* and *TagIn* which are not expressed by capillaries, venules, and only faint by large veins, making them indistinguishable from PCs (Vanlandewijck et al., 2018). Performing further analysis on this population, no subclusters were identified, suggesting that within one tissue type pericytes are very uniform and show little heterogeneity (Vanlandewijck et al., 2018).

Vanlandewijck et al. (2018) went further on to examine whether murine PCs from different tissue samples show heterogeneity, by comparing them to murine lung tissue. Both cell populations expressed conserved markers, such as *Vtn, Higd1b, S1pr3, Mcam, Ifitm1, Baiap3*, and *Ehd3* (Vanlandewijck et al., 2018), suggesting close relation to each other. By performing further differential analysis, they singled out *Anpep* and *Atp13a5* as specific marker for brain PCs. Thus proving that pericytes express heterogeneity *between* different tissue types.

These findings however, can only be understood as assumptions in the intense debate on the definition of PC characteristics (Ferland-Mccollough et al., 2017), as their generally accepted characterization only broadly defines them as cells embedded within the vascular basement membrane (Crisan et al., 2008; Armulik et al., 2011; Ferland-Mccollough et al., 2017) – leaving out the possibility to define different sets of cells as pericytes.

According to Crisan et al. (Crisan et al., 2008, 2012), arterial pericytes express neural/glial antigen 2 (*NG2*) as well as α*SMA*, while capillary pericytes lacked these markers (Crisan et al., 2012; Ferland-Mccollough et al., 2017), again indicating the existence of a heterogeneous PC population rather than uniformity within the same organ. Moreover, Smyth et al. (2018) reported that capillary-associated PCs express *Pdgfrb, NG2, CD13*, and *CD146*, diverging from the previously cited reports (Crisan et al., 2008, 2012; Armulik et al., 2011; Ferland-Mccollough et al., 2017; Smyth et al., 2018; Vanlandewijck et al., 2018).

The reasons for such differing results remain unclear. Whilst Smyth et al. (Smyth et al., 2018) used human brain tissue, Crisan et al. (Crisan et al., 2008) used multiple human tissues from adult and fetal specimen (including the brain), and Vanlandewijck et al. (2018) employed a model of murine brain PCs (Crisan et al., 2008; Smyth et al., 2018; Vanlandewijck et al., 2018).

It remains to be further investigated if the differences originate from a real heterogeneous population, or rather the deviation can be accounted to diverging methodology and models. For that, it should be taken into consideration if pericytes in the different tissues have a common denominator, or whether they should be considered independent cell types of their own in each tissue. Consecutively, a redefinition of already accepted markers and features will need to take place for a deeper and concise PC characterization.

#### Mural Vessel Zonation

Examining murine brain vessel samples, Vanlandewijck et al. (2018) studied the transcriptional identity of mural vessel cells such as pericytes and SMC. *Cnn1* was used as a marker for arterial SMC with a diameter larger than 13 μm. *Acta2* and *TagIn* were used to identify arterial SMCs present in vessels with diameters larger than 8μm. Interestingly, *Acta2* and *TagIn* were absent in capillaries pericytes and venules, and hardly detectable in large veins. Conducting an interspecies comparison, the authors proved that *TagIn* was also markedly expressed in zebrafish brain arteries, suggesting mural cell heterogeneity to be evolutionarily conserved (Vanlandewijck et al., 2018). Lastly, *Abcc9*, which was used as a marker for murine venous SMC and pericytes, was also found in a zebrafish line labeling mural cells in capillaries and veins, backing the conservation mechanism (Vanlandewijck et al., 2018).

Liu and Gomez (2019) reviewed, that an expression pattern *of Myh11, Acta2, Tagln*, and *Myocd* can be utilized to identify SMC, congruent with the findings of Dobnikar et al. (2018). In their study, scRNAseq of murine aorta found the existence of seven subclusters within the designated SMC population, supporting the understanding that SMCs are highly heterogeneous. They further identified that single subclusters are locally expressed, describing *Pde1c* and *Hand2* as marker genes of SMC for the aortic arch region, whilst *Hoxa7* was expressed in the descending thoracic aorta (Dobnikar et al., 2018). These findings suggest that SMCs are not only heterogeneous between different sections of the vascular tree, but also differ within the same vessel.

In stark contrast to the seamless continuum in ECs, mural cells sorted on a trajectory did not follow anatomical directions. When examined for transcriptional relatedness using the SPIN method, pericytes where most closely related to venous SMCs that then neighbored arteriolar SMCs on the trajectory, and lastly arterial SMCs which were closest related to the arteriolar SMCs (Tsafrir et al., 2005; Vanlandewijck et al., 2018). Two discreet subclusters were detected, one formed by pericytes and venous SMCs, that merged gradually by loss of PC markers and gain of venous SMC markers. The other subcluster, separated by an abrupt transition between venous SMC and arteriolar SMC, was formed by arteriolar SMC that merged into arterial SMC by gradually expressing markers denoting them as arterial SMCs. The transition between the two clusters happens abruptly from one cell to another (Vanlandewijck et al., 2018).

#### Key Points

ECs are proven to differ not only between different sections of the vascular tree, but also on a cell-to-cell basis within the same vessel. Capillary ECs are more heterogeneous and express markers according to their surrounding tissue, while large vessels express little heterogeneity and present more conserved markers, independently of their position in the body. Endothelial cells relate to each other on a consistent, seamless trajectory, without expressing distinct, isolated phenotypes. Moreover, key evidences suggest that the gender of the individual also accounts for additional heterogeneity. Contrarily, identification criteria of PCs are not universally accepted, hence comparing results from different authors is hardly possible. Finally, SMCs are highly heterogeneous, express distinct subpopulations, and differ also within the same vessel. However, they appear to not present a confluent trajectory, but rather express distinct isolated subgroups which are confluent within themselves. Limitations to the novel marker gene identification should be kept in mind, as consolidating orthogonal studies confirming the *in silico* findings are yet to be published.

## Pathological Vessel Heterogeneity

It is evident that new vessels are formed physiologically but this phenomenon is also observed in many other pathologies, including cancer (Carmeliet, 2003). Therefore, to fully comprehend pathological vessel heterogeneity, we will first review the initial steps of vessel formation.

In the embryonic stage of development, the formation of the heart and primitive vascular plexus is called vasculogenesis. Postpartum, development, remodeling, and expansion of blood vessels and their network is called angiogenesis (Patan, 2004). Angiogenesis can be distinguished in two main different processes: intussusceptive microvascular growth (Ribatti and Djonov, 2012) and sprouting angiogenesis. Further forms are known, such as vasculogenesis by endothelial progenitor cells and vascular mimicry. Amongst all, sprouting angiogenesis receives most of the attention and is the prime model, on how growing tissues sustain themselves with nutrients and oxygen. This process can be summarized as the formation of endothelial sprouts that denote expansive growth from pre-existing vessels which then form collateral bridges (Carmeliet, 2003). Once the vasculature has reached its maximum extent and supplies all cells, ECs go into quiescence, where they remain for most of the life as a relatively stable cell population (Risau, 1997; Blanco and Gerhardt, 2013). Physiologically, the turnover of quiescent ECs is measured in years (Bergers and Benjamin, 2003). There are some physiological examples for sprouting angiogenesis, such as wound healing (Tonnesen et al., 2000) and the female reproductive cycle (Rizov et al., 2017). Sprouting angiogenesis has also been named one of the hallmarks of cancer, thus underlining, that this physiological process is highjacked in many pathologies.

### Mechanism – Sprouting Angiogenesis

As new vessel formation has been already extensively reviewed, our objective is to illustrate how pathological vessels come into existence and how they differ from their counterparts. Hence, only a summary of the process of neoangiogenesis will be addressed (for further reading see references Carmeliet, 2003; Blanco and Gerhardt, 2013; Minami et al., 2019).

In a physiological setting, neo-angiogenesis is tightly regulated by a balance of pro- and anti-angiogenic signals (Bergers and Benjamin, 2003). Vital for new vessel sprouting is the local production of vascular-specific pro-angiogenic factors, such as vascular endothelial growth factor (VEGF; Blanco and Gerhardt, 2013), which is induced by hypoxic conditions (triggering HIF1 & 2 expression) but also by cytokines, growth factors, hormones, or oncogenes (Dvorak, 2005). VEGF stimulates physiological and pathological angiogenesis in a strict dose-depending manner, creating a gradient that leads the direction (Carmeliet, 2003). It is relevant to mention that on a cellular level, sprouting angiogenesis requires the local break down of the vessel wall, the disintegration of the basement membrane, the change in cellular phenotype, and the invasion of the surrounding tissue (Blanco and Gerhardt, 2013). So-called tip cells that spearhead the sprouts and process cues of the microenvironment to define the route of the new vessel, are responsible for this process which utilizes the help of newly formed filopodia (Gerhardt et al., 2003; Blanco and Gerhardt, 2013). They also create new connections between different sprouts to generate a functional network (Isogai et al., 2003).

**FIGURE 4:**
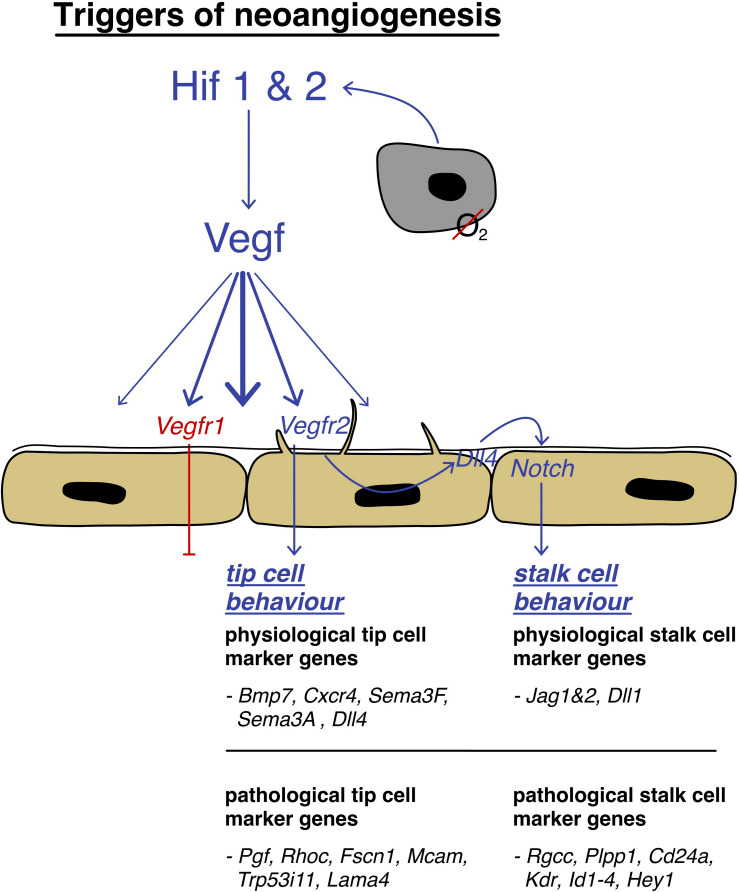
Triggers of neoangiogenesis: schematic depiction of the activation pathway of neoangiogenesis, strongly mediated by HIF1/2 and VEGF and the involvement of tip cells that are leading stalk cells to the hypoxic area by filopodia guidance with their differentially expressed genes.

Tip cells are followed by so-called stalk cells which lack the expression of many filopodia, but are highly proliferative. They establish the adhesions to create a stable inner lining of the newly formed vessel (Blanco and Gerhardt, 2013). Endothelial cells express several relevant cell surface receptors, such as Dll4, VEGFR1, and VEGFR2 – to mention a few relevant ones (Claxton and Fruttiger, 2004; Blanco and Gerhardt, 2013). To accomplish their functions, ECs are highly self-organized and those receptors are of crucial importance. Whilst VEGFR2 activation leads to high kinase activity, VEGFR1 does not trigger an intracellular response to that extension and acts as a decoy receptor preventing exacerbated vessel sprout formation by taking up excess VEGF (Park et al., 1994). Blanco and Gerhardt (2013) showed that VEGFR levels translate directly to Dll4 expression, a ligand that induces Notch signaling in adjacent cells, suppressing the development of a tip cell phenotype, and successively leading to the development of the stalk-like behavior (Blanco and Gerhardt, 2013). This phenomenon is best described by the concept of “lateral inhibition,” a phenomenon well-known from neuronal cells (see Figure 4). This inhibition is vital in organizing appropriate spacing between new sprouts, as several studies have shown that inhibition of DLL4/Notch signaling leads to a dramatic increase in sprouting, vessel branching, and formation of filopodia (Hellström et al., 2007; Lobov et al., 2007; Suchting et al., 2007; Blanco and Gerhardt, 2013). Conversely, it has been shown that Notch gain of function leads to decreased branching (Hellström et al., 2007). Alongside to Notch signaling (Blanco and Gerhardt, 2013), metabolism has shown to have important effects on angiogenesis (De Bock et al., 2013). It was recently discovered that glycolysis has a significant effect on the potency of ECs to acquire tip cell features. As ECs are highly glycolytic and glycolysis is used to cover up to 85% of their energy need, knock-down of the glycolytic activator enzyme PFKFB3 was proven to decrease the length and number of vessel sprouts. *Vice versa*, hypermutation of PFKFB3 leads to unorganized hyper sprouting (De Bock et al., 2013). To summarize, the “battle for the lead” (Blanco and Gerhardt, 2013) is decided by stochastic differences between cells which express VEGFR2 and the local VEGF levels and their metabolic capacity that provides individual cells the advantage over their competitive neighboring cells in acquiring the tip cell phenotype (De Bock et al., 2013). VEGFR2 expressing cells become tip cells, VEGFR1 expressing cells follow a stalk-like behavior (Blanco and Gerhardt, 2013). On a final note, tip and stalk features do not represent a final but rather a dynamic differentiational flux, which can be altered when microenvironmental signals are changing (Bentley et al., 2009; Blanco and Gerhardt, 2013).

### General Differences From Physiological Vessels – Vessel Remodeling

**FIGURE 5:**
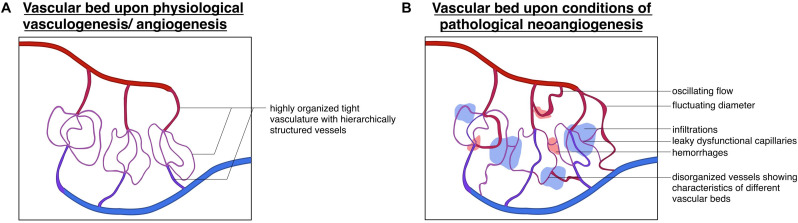
Divergence of physiological and pathological vascular beds: schematic depiction of the **(A)** vascular bed upon the physiological processes of vasculogenesis/angiogenesis displaying a highly organized vessel hierarchy and **(B)** vascular bed upon conditions of pathological neoangiogenesis, showing a highly unorganized structure with (hemorrhagic) infiltrations oscillating flow patterns and fluctuating vessel diameters.

What sets apart the physiological and pathological pattern of vessel formation can easily be summarized into one phrase: “Cancer represents a dysregulation of the body’s normal controlled cellular programs” (Farnsworth et al., 2014). Newly formed tumor vessels lack the tight regulation and hierarchically ordered patterning that can be found in the healthy body. Tumor vessels are heterogeneous, irregularly branched, differ in circumference, are typically enlarged, and often hyperpermeable (Bergers and Benjamin, 2003; Nagy et al., 2006; Carmeliet and Jain, 2011b; Nagy and Dvorak, 2012; Sun et al., 2018). This remodeling process leads to altered EC-PC interaction and abnormal, oscillating blow flow (Bergers and Benjamin, 2003; see Figure 5). Expression of different angiogenetic growth factors leads to distinct patterns of angiogenesis, which can be observed in diverse tumors (Farnsworth et al., 2014). As observed in some tumors originating from the lung, colon, or brain, these can show a lower vessel density then the normal healthy tissue (Eberhard et al., 2000; Bergers and Benjamin, 2003). However, vessel density cannot be taken as a predictor for the aggressiveness of the tumor, as grade I pilocytic brain tumors are highly angiogenic, but are slow-growing and do not metastasize (Tomlinson et al., 1994; Bergers and Benjamin, 2003), whilst tumors such as chondrosarcomas are very aggressive but show a very low vessel density (Brem et al., 1972; Bergers and Benjamin, 2003). Certain tumor entities rely on the mobilization of endothelial progenitor cells, known as vasculogenesis (Lyden et al., 2001). Others, such as low-grade astrocytomas utilize a completely different approach, connecting themselves up to pre-existent vessels in a poorly understood process called vessel co-option. Hence, they are generally considered non-angiogenic tumors (Bergers and Benjamin, 2003).

Growth of tumor vasculature is not only driven by VEGF, but a dysregulated ensemble of many factors, such as angiopoietins, platelet-derived growth factor, and transforming growth factor families (Carmeliet and Jain, 2011a). As a result of this uncontrolled angiogenic process, mechanisms that normally ensure strong and occluding endothelial cell junctions are lost. Consecutively, tumor ECs show excessive permeability which can be, amongst other things, explained by the low EC to perivascular cell ratio. Pericytes that usually take part in vessel regulation are not only scarce in tumor-associated vessels, but also shown to be mutated, leading to vascular malformations (Morikawa et al., 2002; Bergers and Song, 2005; Viallard and Larrivée, 2017). Additionally, tumor vessels’ basement membrane is commonly found altered (Baluk et al., 2003), contributing to leaky vessels. Tumor vessels lack the typical cobblestone-like lining of ECs which can occasionally be found multi-layered. A vascular tree cannot be distinguished, and their arterial or venous identity is lost. Nevertheless, shunts can be found (Potente et al., 2011). In pathological angiogenesis, large transient mother vessels are the first to form, arising from capillaries (Farnsworth et al., 2014). Those mother vessels are characterized by their large diameter as well as their thin and permeable walls. These vascular malformations later obtain an irregular coat of smooth muscle fibers and reassemble abnormally large veins (Nagy et al., 2010). Although tumor vessels remodeling is often characterized by the physical repositioning of the cellular components, molecular alterations often accompany the process (Farnsworth et al., 2014). Illustrating this concept, we present the genetic differences of marker genes in physiological and pathological tip and stalk cells in Figure 4 (Hofmann and Luisa Iruela-Arispe, 2007; Strasser et al., 2010; Rohlenova et al., 2020).

### Recent Findings in Pathological Vessel Heterogeneity

#### Retinal Vessel Sprouting in a Murine Model

Recently, a study focused on the differences between healthy choroidal ECs and choroidal neovascularization (CNV), in the context of wet age-related macular disease (Rohlenova et al., 2020). Utilizing a pre-clinical murine model of laser-induced CNV they obtained tissue which was consecutively sequenced at single-cell resolution. When comparing healthy murine ECs and ECs from eyes that underwent laser treatment they observed the existence of a distinct subcluster in CNV. Further gene signature analysis proved the existence of proliferating ECs and tip cells, but also three new EC populations. One population showed signatures of transitioning from a postcapillary venule (PCV) to an angiogenic phenotype, whilst the other two populations were termed immature and maturing ECs (Rohlenova et al., 2020). Interestingly, proliferating cells showed increased expression of the transcription factor *Trp53*. Tip cells upregulated the disease restricted angiogenic factor *Pgf* and showed that the transcription factor *Tgif1* was involved in EC designation, whilst immature ECs did not present specific upregulation of marker genes, but more unspecific patterns of activation markers. The authors also showed that the transcription factors *Smad1* and *Sox4* are involved in EC development (Rohlenova et al., 2020). Maturing cells, which are also termed phalanx cells, overexpressed a Notch signaling signature. Rohlenova et al. (2020) showed that activated and transitioning PCV cells upregulated *Nr2f2*, which is in line with previous reports (Jeong et al., 2017; Rohlenova et al., 2020). When performing pseudotime analysis, Rohlenova et al. (2020) showed the existence of a seamless trajectory within this additional cluster which starts with activated PCV cells that evolve to CNV transitioning cells, and are followed by immature EC cells, tip cells, and mature phalanx cells. These findings indicate that neovascularization might originate in PCVs as previously predicted by morphological evidences (Folkman, 1982; Rohlenova et al., 2020). The same study further evaluated commonly shared markers, which are also overexpressed in different tumor ECs originating in different tissues (Rohlenova et al., 2020). When performing combined differential analysis between their data set of CNV and a previously generated data set on lung tumor ECs (Goveia et al., 2020), the authors found proliferation, hypoxia, and extracellular matrix formation pathways to be commonly upregulated (Rohlenova et al., 2020) and identified *Aplnr* as a congruent marker between these EC populations. *Aplnr* is an angiogenic and vasculoprotective gene that regulates EC metabolism (Rohlenova et al., 2020). Importantly, when pseudo-time analysis was performed on lung tumor ECs that were previously collected by an affiliated author (Goveia et al., 2020), the same trajectory was found as in Rohlenovas’ (Rohlenova et al., 2020) CNV-EC samples. Lung tumor ECs that expressed the gene pattern of veins were at the start of the trajectory, which then changed their gene expression profile, differentiating to PCV immature ECs. These cells then further developed into tip cells, losing their previous marker genes. Along the pseudo time trajectory, they then express markers of neo-phalanx cells, and lastly markers of activated arteries. These findings indicate that the neoangiogenic process follows a conserved pattern of stages in at least two different tissue types (Goveia et al., 2020; Rohlenova et al., 2020).

#### Pathological Vessels in Human Lung Carcinoma

Recent studies focused on the comparison of EC heterogeneity of non-small lung cancer to the healthy peri-tumoral vasculature. Goveia et al. (2020) sequenced the transcriptome of eight different human lung cancer EC specimens on a single-cell level and compared the transcriptome to their healthy counterparts. As the vasculature is rather quiescent under normal circumstances, the authors only found the transcriptome patterns of classical angiogenic phenotypes in tumor samples involving tip and proliferating ECs (the latter only being sparsely transcribed). Tip cells expressed genes involved in VEGF signaling, EC migration matrix remodeling, and the disease-specific molecule *pgf* [which was also detected by Rohlenova et al. (2020) in CNV] (Goveia et al., 2020). Interestingly, the authors describe the existence of an immature EC phenotype, which reassembled stalk-like cells, showing up-regulation of gene related to vessel maturing, vessel barrier integrity and notch signaling (Goveia et al., 2020). Also, they found another tumor restricted phenotype, which they termed “activated post-capillary veins,” as these ECs upregulated immunomodulatory factors and reassembled features of high endothelial venules (Goveia et al., 2020). Besides detecting the transcriptome signature of previously described EC subpopulations, Goveia et al. (2020) identified two novel capillary phenotypes, which were suspected to be induced by tumor-derived cytokines. In accordance with their gene signature, they were termed “scavenging ECs” (scavenging receptors, macrophage associated genes, and antigen processing) and “activated capillaries” (activation markers). As Zhao et al. (2018) described the phenomenon of tip cells showing different markers in different tumor models, Goveia et al. (2020) made use of a mouse model to further cross-validate their findings. In this analysis, the authors found that the sparsely detected proliferating ECs were more abundant, and that these ECs could be traced back to faster-growing murine carcinomas and a possibly different type of tumor vascularization (Goveia et al., 2020). Moreover, the presence of neophalanx cells, an even more mature angiogenic cell population that expressed capillary and arteriole markers (Goveia et al., 2020), was observed. Surprisingly, the authors found the presence of a previously unknown population, which upregulated tip EC, and VEGF-associated basement and collagen remodeling markers, later named “breach” and “pre-breach” cells (Seano et al., 2014).

## Liver Vasculature

This review section aims to take a closer look at the development of the hepatic vasculature in both its embryonic and adult stages. Furthermore, this section also addresses how primary tumors may metastasize into the liver and how metastatic cells can present different behaviors within the hepatic microenvironment. A better understanding of these points is crucial to the development of new therapeutic options in the treatment of liver metastases.

### Normal Hepatic Vasculature Development and Sinusoids’ Microenvironment

The liver is considered the largest mass of glandular tissue in the human body and its development starts at the beginning of the fourth embryonal week (Pawlina, 2020). At the 10th-week hepatic vasculogenesis starts. Interestingly, the hepatic vasculature arises from different embryological layers. Intra-hepatic arteries are seen first in the 10th-week in the central portal tracts and in the 15th-week also in the peripheral part of the liver. They are formed by neoangiogenesis, this process beginning in the perihilar region and advancing toward the peripheral region (Gouysse et al., 2002; Pawlina, 2020). The sinusoids differentiate from capillary vessels of the septum transversum, whilst portal veins differentiate from vitelline veins (Gouysse et al., 2002). The intraportal vessels differentiate from mesenchymal precursor cells (Gouysse et al., 2002). Their corresponding endothelial cell subpopulations exhibit a high degree of cellular differentiation, especially those forming the sinusoids (Gouysse et al., 2002).

Vascular development and differentiation during organogenesis are driven by different aspects like cytokine activity (e.g., by VEGF, Interleukins), and the cellular microenvironment composition, including components of the extracellular matrix-like integrins (Risau, 1997). As already mentioned, the angiogenic profile of the endothelial cells will differ depending on their belonging tissue (Rafii et al., 2016). From the 5th- to 10th-week of embryonal development the existing vessels are derived from pre-existing vessels with a low differentiation status (Gouysse et al., 2002). A low differentiation status means that the endothelial cells are not highly specialized as are endothelial cells from hepatic sinusoids, which exhibit certain structural and functional characteristics like cytoplasmatic fenestration and a gain of differentiation markers like CD4 (Gouysse et al., 2002). ECs of large embryonic vessels like the precursors of portal veins express CD34 and are surrounded by a tenascin-rich matrix, whereas the precursors of sinusoids, such as the capillary vessels of septum transversum, still behave like a continuous endothelium and are generally surrounded by a laminin-1-rich matrix (Gouysse et al., 2002). As previously noticed, pericytes surround vessels and are externally located on their wall. These cells are also considered mesenchymal stem cells and can be detected by their CD146 expression (Shenoy and Bose, 2018). In mice, hepatic pericytes can be differentiated in two main types: (i) a subpopulation with myogenic features and (ii) a second population with fibrogenic behavior. Whilst myogenic pericytes form multinucleated myotubes, fibrogenic pericytes develop into myofibroblasts (Shenoy and Bose, 2018). In humans, vascular hepatic pericytes can be differentiated according to their cluster of differentiation. Whilst pericytes surrounding the portal vein and hepatic artery express consistently CD146, pericytes in other hepatic areas express low levels of CD146 (Strauss et al., 2017).

As discussed in “Pathological vessel heterogeneity,” vasculogenesis is seen in the embryonal development. During adulthood, hepatic vascularization is mainly triggered by lower blood flow which leads to an increase in VEGF release and consecutive proliferation of hepatic ECs. In this scenario, new collateral vessels are formed (Dirscherl et al., 2020). These ECs show specific differentiation markers like CD4, which is specific for the discontinuous endothelium of sinusoids. The sinusoidal hepatic endothelial cells also take an important role in pathological conditions such as liver fibrosis and cirrhosis (DeLeve and Maretti-Mira, 2017). Moreover, liver sinusoidal endothelial cells can release anti-inflammatory cytokines, like TGF-β which can inhibit the inflammatory response in these conditions (Ni et al., 2017).

Cancer and its tumor-associated microenvironment not only affects tumor progression but also has a high impact on the development of metastases (Zhang et al., 2018). The hepatic microenvironment is composed by a complex and interconnected group of highly specialized cells. Surrounding the liver sinusoids we can observe Kupffer cells, which are a part of the mononuclear phagocytotic system, and are involved in the final elimination of erythrocytes and in the recycling of its fragments and ferritin (Pawlina, 2018). We also observe hepatic stellate cells in the liver parenchyma. These cells can store vitamin A and, in pathologic conditions, like chronic inflammation, are also able to transdifferentiate into myofibroblasts and further synthesize collagen (Pawlina, 2018).

It is important to understand the interaction between the tumor or metastases and the microenvironment, to develop new therapeutic approaches. Recent findings have shed a light on the underlining mechanisms involving hepatic stellate cells and angiogenesis (Dirscherl et al., 2020). It has been shown that hepatic stellate cells have the ability to sense hypoxia and subsequently release VEGF which further increases angiogenesis. Additionally, an increase of vWF and CD34-positive endothelium was seen in hypoxia-exposed liver tissue (Dirscherl et al., 2020). Thus, these in vitro results show an important interaction between the hepatic microenvironment and angiogenesis which might, in a transformed, malignant scenario, promote tumor growth.

**FIGURE 6:**
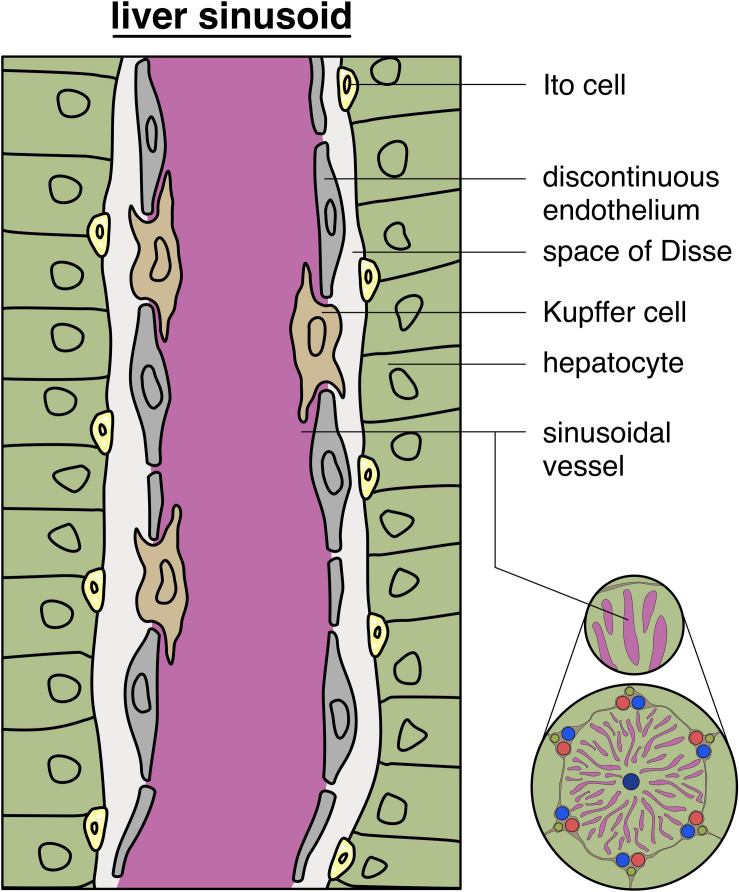
Structure of liver sinusoids: schematic depiction of a typical liver sinusoid showing a discontinuous endothelium with intercalated Kupffer cells in the vessel wall, with additional glimpse of the hepatic lobule (on the bottom, right).

The afferent blood supply of the adult liver is made up mainly by the portal vein and the hepatic artery (Pawlina, 2018). At the *porta hepatis*, both enter the liver and supply the capillary networks. Most of the liver’s blood supply comes from the portal vein (approx. 75%), with low oxygen levels, as it has previously collected the blood from the digestive tract, pancreas, and spleen (Pawlina, 2018). Derived from intra-hepatic mesenchymal precusors, interlobular vessels, accompanied by bile ducts, form the portal triad, draining their blood into the sinusoids (Gouysse et al., 2002). The sinusoids are highly specialized capillary vessels that transport arterial and venous blood (see Figure 6). In the definition of a classic liver lobule, the blood flows from the portal vein and hepatic artery right into a sinusoid. The sinusoids send blood into the central vein (Pawlina, 2018). Hepatic sinusoidal endothelial cells show a fenestrated endothelium, however, during inflammatory conditions this fenestra are reduced in size, and the amount of endothelial cells and the basement membrane becomes discontinuous (Ni et al., 2017). The central vein further enters a sub lobular vein which finally flows into the inferior vena cava. Sinusoids show a discontinuous basal membrane and big fenestrae without diaphragms within the ECs (Pawlina, 2018).

#### Hepatic Regeneration

The hepatic tissue exhibits the unique capability to regenerate itself after injury (Michalopoulos, 2007; Mao et al., 2014; Tao et al., 2017). After toxic damage or loss of hepatic tissue, the liver enters, a still not completely understood, process that cumulates in its restorage in size, via hyperplastic growth (Mao et al., 2014). Starting at the portal field (hepatic lobules), a front of hepatocytes enters into mitosis advancing toward the central vein (Rabes, 1977). Interestingly, all hepatocytes undergo mitosis, contrary to other regenerative scenarios, such as in the skin or intestine, where a group of proliferating stem cells creates the mass of new cells (Michalopoulos, 2007). This peculiar regenerative behavior leads to the observation that the hepatocyte plates grow to almost twice their normal size (Michalopoulos and DeFrances, 1997), with the liver reaching its former volume after 8–15 days past injury (Michalopoulos, 2007). Basically, the hepatic regenerative process can be summarized in three stages. First, the activation of more than 100 genes which are generally silenced during homeostasis can be observed. Surprisingly, IL-6 is suggested to be responsible for approximately 40% of this regulatory mechanism. Second, a massive proliferation wave (also called progression phase) begins and the majority of all hepatocytes enters into mitosis. Crucial mitogenic factors for this phase are HGF, TGF-α, EGF, and HB-EGF. Finally, homeostasis is achieved and the process is terminated. Although this final process still remains poorly understood, recent findings suggested that TGF-β1 might be directly involved in its conclusion (Mao et al., 2014). Strikingly, all these phases take place whilst the liver maintains its homeostatic functions (Michalopoulos, 2007). This astonishing performance gives rise to different medical practices, such as dividing graft organs between recipients or resecting large metastatic areas. In this specific oncological situation, it is imperative that after the resection of the metastatic site the future remnant liver can cope with its normal homeostatic functions and hemodynamic stress. To achieve that, the use of hepatic portal vein embolization (PVE) has been employed since the 1990’s (Makuuchi et al., 1990). Briefly, the procedure redirects the hepatic blood flow to segments of the future liver remnant which ultimately results in hepatic hypertrophy. Recent RNA-seq profiling data of liver regeneration models contributed to the identification of a molecular signature and regenerative signaling pathways involved in hepatic regeneration in rats, upon surgical procedures (Colak et al., 2020a, b). The authors’ analyses evidenced transcriptomic changes in genes associated with cell cycle (e.g., *TP53, RB1, CCND1*), transcription factors (e.g., *Myc, E2F1, FOXM1*), DNA replication regulators (e.g., *EZH2, CDKN1A, RRM2*), G1/S- transition regulators (e.g., *RABL6, CCND1*), growth factors and cytokines (e.g., *CSF2, HGF, IL-6*). Nevertheless, the participation and active cross-talking between cells of the hepatic milieu, like hepatic stellate cells, has been already confirmed (Dirscherl et al., 2020). Notably, also ECs can directly contribute to hepatic regeneration, as *in vivo* data generated with Id-1-deficient mice show, which present reduced number of liver sinusoidal EC, demonstrated that upon hepatic damage this population releases angiocrine factors, such as Wnt2 and HGF, triggering hepatocyte proliferation and liver regeneration (Ding et al., 2010).

### Mechanisms of Hepatic Tumor Dissemination

#### Intra-Hepatic Tumor Angiogenesis

As reported in “Mechanism – Sprouting Angiogenesis” and “General Differences From Physiological Vessels – Vessel Remodeling,” angiogenesis is a tightly regulated process that is highjacked in an unorganized manner by the tumor environment. Neoangiogenic vessels that develop during carcinogenic processes are different from those that are generated in the physiological process of angiogenesis (Jain, 2014). It has been previously shown that one possible reason for this difference is that tumor endothelial cells are exposed to extremely high amounts of VEGF from tumor cells and tumor-associated fibroblasts (Jain, 2014). Additionally, angiopoietin, PDGF-B, and TGF-ß are also responsible for the development of less functional vessels in tumors (Viallard and Larrivée, 2017). Tumor blood vessels show a very atypical morphology, the vessels are dilated and disorganized and are leaky (Jain, 2014; Viallard and Larrivée, 2017). This leakiness can cause edema due to plasma extravasation which can significantly slow down blood flow as erythrocytes concentrated and interstitial hypertension increases (Jain, 2014). Nevertheless, tumor endothelial cells are also highly glycolytic, therefore they generally differentiate into tip cells that are responsible for sprouting, rather than into stalk cells, which give vessels stability. This deficit in stability and aberrant architecture leads to an unordered and less effective vascularization and consequently higher hypoxia levels and worse tumor nutrition (Jain, 2014).

#### Heterogeneity Within Metastases

Several different types of solid tumors and their metastases overexpress one or more types of growth factors of the VEGF family which help them to achieve their vascular supply. The sprouting of vessels starts at a very early carcinogenic stage, generally when tumors reach 2–3 mm^3^ in size (Vasudev and Reynolds, 2014). This suggests that an anti-angiogenic treatment can affect most types of solid tumors and their metastases (Vasudev and Reynolds, 2014; Itatani et al., 2018). Conflicting, it has been already proven that some solid tumors and their metastases do not benefit from anti-angiogenic therapy (Hurwitz et al., 2004). According to the IMPOwer 150 (NCT02366143) study, patients from all subgroups with metastasized non-squamous NSCLC, which were previously defined by their PD-L1 expression, benefited from additional treatment with bevacizumab. This is of high clinical relevance, as the first line monotherapy with PD-L1 inhibitors is actually limited for patients with a high PD-L1 expression. Interestingly, those synergistic effects could not be observed in other two recent clinical trials [IMPOwer132 (NCT02657434) (Papadimitrakopoulou et al., 2018) and IMPOwer130 (NCTT02367781) (West et al., 2019)], highlighting the complex hepatic environment, where traditional VEGF inhibition in addition to immune-oncological therapy show clear benefits. These results further support new treatment options with PD-L1 inhibitors for patients with hepatic metastatic disease, even for patients who exhibit low PD-L1 expression (Socinski et al., 2018).

As patients with metastases can often not be treated curatively by surgery, therapeutical options that slow down tumor growth are of extreme importance. In addition to VEGF blockade, the use of tyrosine kinase inhibitors show positive effects on advanced hepatocellular carcinoma and advanced pancreatic neuroendocrine tumors, and is a promising option in pancreatic adenocarcinoma (Vasudev and Reynolds, 2014; Daneshmanesh et al., 2018).

These varying results denote the need to find new biomarkers or other prognostic features to predict the response of patients to an anti-angiogenic therapy. New biomarkers could also improve the further understanding why metastases respond differently to anti-angiogenic therapy. Colorectal cancer is well-known to often spread into the liver due to the intestinal drainage system. Also, lung, brain, pancreatic and cervical tumors present this chemotactic predilection (Yachida et al., 2010; Assoun et al., 2017; Tewari et al., 2017). Besides drainage, there are other mechanisms which can favor hepatic metastatic colonization. It has been already shown that tumor cells favor certain types of endothelial interaction which would explain the reason they metastasize into certain organs more often than into others (Nguyen et al., 2009; Dasgupta et al., 2017). Analysis of the genetic determinants involved in this process revealed the participation of genes related to cell motility, epithelial-to-mesenchymal transition, extracellular matrix degradation, and bone marrow progenitor mobilization, such as *TWIST1, SNAI1, SNAI2, MET, ID1 KISS1, miR-126, miR-335, DARC*, and *GPR56* (Nguyen et al., 2009).

Regarding colorectal cancer liver metastasis, genotyping of tumor specimens is becoming a standard diagnostic practice, with the evaluation of several relevant oncogenes, such as *KRAS, BRAF, PIK3CA*, and *NRAS* (Huang et al., 2018; Sagaert et al., 2018). This information allows the prediction of therapy response and can set patient’s prognosis. Interestingly, some degree of inter-metastatic heterogeneity is observed in some patients. In a study evaluating KRAS mutational status, it was observed that 6.8% of the evaluated metastatic sites differ between themselves. The same study also observed heterogeneity in the status of BRAF mutations (Turajlic et al., 2020).

#### Different Mechanisms of Blood Irrigation

It is already well described that liver metastases can present different growth patterns (Frentzas et al., 2016; van Dam et al., 2017; Hoppener et al., 2019). The most prevalent type in pre-existing colorectal cancer liver metastases after chemotherapy in combination with bevacizumab is the desmoplastic growth pattern – where the tumor cells are surrounded by a desmoplastic rim, composed mainly by fibroblasts and immune cells (Frentzas et al., 2016; van Dam et al., 2017; Hoppener et al., 2019). The desmoplastic growth pattern utilizes neoangiogenesis to supply tumor cells with nutrients and oxygen (Frentzas et al., 2016). Generally, these metastases produce high levels of VEGF which then induce vessel sprouting. Another hepatic metastatic growth pattern, which also uses the mechanism of neoangiogenesis to obtain its blood supply, is the pushing growth pattern (Hoppener et al., 2019). In this growth pattern, which accounts for approximately 5% of the observed patterns, also in pre-existing colorectal cancer liver metastases after chemotherapy in combination with bevacizumab, the tumor cells push the healthy liver parenchyma aside, without building a desmoplastic rim (Frentzas et al., 2016; van Dam et al., 2017).

**FIGURE 7:**
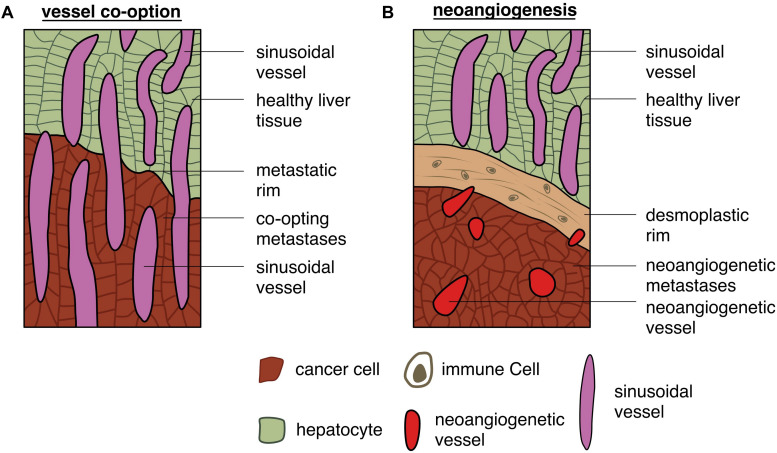
Different capabilities of gaining access to the vasculature: schematic depiction of **(A)** tumor cells gaining access to vessels by co-option and **(B)** tumor cells accessing vessels by triggering neoangiogenesis.

Yet, according to Frentzas et al. (2016), the second most prevalent metastatic growth pattern, accounting for approximately 45% of the detected growth patterns, is the replacement type (see Figure 7; Frentzas et al., 2016; van Dam et al., 2017; Galjart et al., 2019). In this situation metastases access their blood supply via co-option, meaning that tumor cells receive oxygen and nutrients from pre-existing vessels, like e.g., the hepatic sinusoids (van Dam et al., 2017). Interestingly, the replacement growth pattern increases from approximately 55% in untreated patients to approximately 85% in recurrent liver metastases after treatment with chemotherapy in combination with bevacizumab (Frentzas et al., 2016). Nevertheless, tumor hepatic growth patterns can present a degree of heterogeneity with patients exhibiting hepatic metastatic sites with different growth patterns (Frentzas et al., 2016). This can be considered one reason why patients with metastatic colorectal cancer respond in an unpredictable way to anti-angiogenic therapy, as the metastatic growth pattern is usually not determined in patients (van Dam et al., 2017). As expected, patients with desmoplastic growth pattern respond better to an anti-angiogenic therapy than patients with the replacement type, relying on co-option (Hoppener et al., 2019). Nevertheless, what makes liver metastases grow in one or another histological growth pattern is still a central question that needs to be further investigated.

Besides the histopathological growth patterns other markers that indicate the response to anti-angiogenic therapy have been already described (van Dam et al., 2017; Incio et al., 2018). Previously, a study has shown that high levels of circulating VEGF-A predicts a survival benefit for anti-angiogenic therapy in patients with metastatic breast and gastric cancer (Vasudev and Reynolds, 2014). This is justified as those tumors will respond better to anti-angiogenic therapy. Further exploring this scenario, Ma et al. (2019) investigated the impact of VEGF-A in primary liver cancers, showing that a high level of VEGF-A expressed by malignant cells leads to a more divers tumor microenvironment which was correlated to a significantly worse overall survival. Two other factors that cause a resistance to anti-angiogenic therapy are IL-6 and FGF-2 that are upregulated under obesity conditions (Incio et al., 2018). Frequently, acquired resistance to anti-VEGF therapy is observed in a significative fraction of patients (Hurwitz et al., 2004). However, a fraction of patients shows innate, intrinsic resistance to anti-angiogenic therapy. This alteration in therapy response also echoes to the different types of growth patterns, as changes from the desmoplastic to replacement type can be observed in patients undergoing anti-angiogenic treatment (van Dam et al., 2018; Hoppener et al., 2019).

Tumor cells heterogeneity is not the only variable which has been described as a possible mechanism that could influence anti-angiogenic therapy resistance. The host organ microenvironment, especially the immune cells, have also been suggested to play a crucial role in in the process of metastasis (Quail and Joyce, 2013; Dagogo-Jack and Shaw, 2018). Macrophages, which usually are critical effector cells in immune response, can support tumor progression (Quail and Joyce, 2013). It has been already shown that tumor-associated macrophages release tumor-derived CSF-1 and macrophage-derived EGF through a paracrine manner, further promoting therapy resistance (Quail and Joyce, 2013). Therefore, CSF-1 might be a new possible target in oncology. Yet, the underlying mechanisms which influence macrophages phenotype switch from a tumor-suppressing to a pro-tumorigenic type are still unclear. However, it is presumed that conditions within the tumor microenvironment, like hypoxia, might cause phenotypical changes (Jain, 2014). High levels of VEGF and endothelin-2 serve as chemoattractants and could be responsible for the recruitment of these cells to hypoxic regions, leading to their correlation with neoangiogenesis and invasion (Quail and Joyce, 2013; Albini et al., 2018). Lastly, regarding tumor microenvironment cells, it is important to reinforce the participation of tumor-associated fibroblasts in the promotion and growth of malignant cells and metastases (Quail and Joyce, 2013). Normal fibroblasts instead promote the growth of healthy endothelial cells and suppress the growth of tumor cells (Quail and Joyce, 2013). Currently, the exact origin of tumor-associated fibroblasts is controverse. It has been suggested that they arise from the endothelial-to-mesenchymal transition (EMT). *In vivo* mouse experiments with melanoma and neuroendocrine cells showed that the tumor-associated fibroblasts are derived from endothelial cells, accumulating in the tumor microenvironment and are activated by several growth factors and cytokines, which ultimately support they cellular turn over (Quail and Joyce, 2013).

#### Key Points

We can recapitulate that angiogenesis is a crucial aspect in the process of metastasis. Many tumors metastasize especially in the liver, where they are confronted with a highly specialized vascular system. The tumor cells instrumentalize different mechanisms to gain access to the blood system. One of the most common is neoangiogenesis, which is driven mainly by VEGF. The metastases are highly dependent on nutrition via the blood vessels which has made neoangiogenesis an interesting target for anti-angiogenetic therapy. Unfortunately, thanks to their heterogeneity, tumors are able to adapt to severe changes in their environment, evidencing the current limitations of anti-angiogenetic therapeutical approaches.

## Conclusion and Future Perspectives

This review summarizes the main aspects involved in developmental and pathological angiogenesis. From the start of embryogenesis to the establishment and maintenance of the human mature adult body, vascularization is vital for all processes. Not long ago, the extensive lack of information regarding the molecular signature of the vascular system started to be revised and filled by elegant, well-designed studies which helped us to better comprehend the complexity of this system. As described in this review, new and advanced molecular techniques, such as single-cell sequencing certainly brought relevant missing pieces of information, which are used not only to deepen our knowledge on molecular mechanisms underlying vascular physiology and pathogenesis, but also to generate new promising therapeutical approaches such as EC metabolic inhibition and tumor-vessel normalization (Schoors et al., 2014; Cantelmo et al., 2016). Those scientific insights are crucial to help us overcome the current setbacks observed in e.g., vascular regeneration and anti-angiogenic therapy. The concepts proposed at the beginning of the angiogenesis research by Judah Folkmann in 1971 (Folkman, 1971), which were heavily criticized and not acknowledged at that time, have never been so extensively tested and recognized. Therefore, the further uncovering and comprehension of the human endothelial and mural cell heterogeneity and their involvement in disease at the molecular and metabolic level, are decisive factors to improve future therapeutic strategies.

## Author Contributions

JF and CJ drafted the manuscript. TD and L-CC conceptualized the review article. JF designed the figures. All authors revised and discussed the manuscript.

## Conflict of Interest

The authors declare that the research was conducted in the absence of any commercial or financial relationships that could be construed as a potential conflict of interest.

## Abbreviations

miR-126: microRNA 126
A2m: alpha-2-macroglobulin
Acta2, actin alpha 2: smooth muscle
Alk1: ALK receptor tyrosine kinase 1
Anpep, alanyl aminopeptidase: membrane
Aplnr: apelin receptor
aSMA: alpha smooth muscle actin
Atp13a5: ATPase 13A5
Baiap3: BAI1 associated protein 3
BMEC: bone marrow endothelial cells
Bmx: BMX non-receptor tyrosine kinase
BRAF, B-Raf proto-oncogene: serine/threonine kinase
CCND1: cyclin D1
CD13: cluster of differentiation 13
CD146: cluster of differentiation 146
CD31: cluster of differentiation 31
CD34: cluster of differentiation 34
CD34: cluster of differentiation 34
CD36: cluster of differentiation 36
CD4: cluster of differentiation 4
CD45: cluster of differentiation 45
Cdh5: cadherin 5
CDKN1A: cyclin dependent kinase inhibitor 1A
Cldn5: claudin 5
Clec4g: C-type lectin domain family 4 member G
Cnn1: calponin 1
CNV: choroidal neovascularizations
CSF-1: colony stimulating factor 1
CSF2: colony stimulating factor 2
Cspg4: chondroitin sulfate proteoglycan 4
Cxcl1: C-X -C motif chemokine ligand 1
Cytl1: cytokine like 1
DARC: Duffy antigen chemokine receptor
Depp: DEPP1 autophagy regulator
Des: desmin
Dll4: delta like canonical notch ligand 4
Dnase113: deoxyribonuclease 113
E2F1: E2F transcription factor 1
ECs: endothelial cells
Efnb2: ephrin B2
EGF: epidermal growth factor
Ehd3: EH domain containing 3
Emcn: endomucin
EPAS1: endothelial PAS domain protein 1
EVP: endovascular progenitor cell
EZH2: enhancer of zeste 2 polycomb repressive complex 2 subunit
Fabp4: fatty acid binding protein 4
Fam167b: family with sequence similarity 167 member B
Fbln5: fibulin 5
Fcgr2b: Fc fragment of IgG receptor IIb
FGF-2: fibroblast growth factor 2
Flt4: Fms related receptor tyrosine kinase 4
FOXM1: forkhead box M1
Gata5: GATA binding protein 5
GPR56: G protein-coupled receptor 56
Hand2: heart and neural crest derivatives expressed 2
HB-EGF: heparin binding epidermal growth factor
hBFGF: human basic fibroblast growth factor
hEGF: human epidermal growth factor
Hey1: hes related family BHLH transcription factor with YRPW motif 1
Hey2: hes related family BHLH transcription factor with YRPW motif 2
HGF: hepatocyte growth factor
HIF1: hypoxia inducible factor 1
HIF2: hypoxia inducible factor 2
Higd1b: HIG1 hypoxia inducible domain family member 1B
Hoxa7: homeobox A7
Ifitm1: interferon induced transmembrane protein 1
Il33: interleukin 33
IL-6: interleukin 6
Il6st: interleukin 6 signal transducer
Itgbl1: integrin subunit beta like 1
Itm2a: integral membrane protein 2A
Jag1: jagged canonical notch ligand 1
Junb, JunB proto-oncogene: AP-1 transcription factor subunit
Kdr: kinase insert domain receptor
KRAS, KRAS proto-oncogene: GTPase
Krtdap: keratinocyte differentiation associated protein
Lcn2: lipocalin 2
Lgr5: leucine rich repeat containing G protein-coupled receptor 5
Ly6a: stem cells antigen-1
Mcam: melanoma cell adhesion molecule
MET, MET proto-oncogene: receptor tyrosine kinaseID1 KISS1
Mfsd2a: major facilitator superfamily domain containing 2A
miR-335: microRNA 335
Myc: Myc proto oncogene
Myh11: myosin heavy chain 11
Myocd: myocardin
NG2: neuron-glial antigen 2
Npr3: natriuretic peptide receptor 3
Nr2f2: nuclear receptor subfamily 2 group F member 2
Nr4a2: nuclear receptor subfamily 4 group A member 2
NRAS, NRAS proto-oncogene: GTPase
Nrp1: neuropilin 1
PCs: pericytes
PCV: postcapillary venules
Pde1c: phosphodiesterase 1C
PDGF: platelet derived growth factor
Pdgfra: platelet derived growth factor receptor alpha
Pdgfrb: platelet derived growth factor receptor beta
Pecam1: platelet and endothelial cell adhesion molecule 1
PFKFB3: 6-phosphofructo-2-kinase/fructose-2,6-biphosphatase 3
Pgf: placental growth factor
Pglyrp1: peptidoglycan recognition protein 1
PIK3CA: phosphatidylinositol-4,5-bisphosphate 3-kinase catalytic subunit alpha
PVE: portal vein embolization
RABL6, RAB: member RAS oncogene family like 6
Rad54b: RAD54 homolog B
RB1: retinoblastoma 1
RRM2: ribonucleotide reductase regulatory subunit M2
S1pr3: sphingosine-1-phosphate receptor 3
SCA-1: stem cells antigen-1
scRNAseq: single cell RNA sequencing
Sema3g: semaphorin 3G
Slc16a1: solute carrier family 16 member 1
Slc2a1: solute carrier family 2 member 1
Slco1c1: solute carrier organic anion transporter family member 1C1
Smad1: SMAD family member 1
SMC: smooth muscle cell
SNAI1: snail family transcriptional repressor 1
SNAI2: snail family transcriptional repressor 2
Sox4: SRY-box transcription factor 4
Sox9: SRY-box transcription factor 9
SPIN: sorting points in neighborhood
Ssh2: slingshot protein phosphatase 2
Stab2: stabilin 2
Tagln: transgelin
Tfrc: transferrin receptor
TGF- α: transforming growth factor α
TGF- β: transforming growth factor β
TGF- β 1: transforming growth factor β 1
Tgif1: TGFB induced factor homeobox 1
Tie1: tyrosine kinase with immunoglobulin like and EGF like domains 1
Tmem100: transmembrane protein 100
Tmem132e: transmembrane protein 132E
TP53: tumor protein 53
Trgj1: T cell receptor gamma joining 1
Trim29: tripartite motif containing 29
Trp53: tumor protein P53
TWIST1: twist family BHLH transcription factor 1
Ush1g: USH1 protein network component sans
Vcam-1: vascular cell adhesion molecule 1
VE-cadherin: vascular endothelial cadherin
VEGF: vascular endothelial growth factor
Vegfc: vascular endothelial growth factor C
VEGFR1: vascular endothelial growth factor receptor 1
VEGFR2: vascular endothelial growth factor receptor 2
VEGFR3: vascular endothelial growth factor receptor 3
Vtn: vitronectin
vWF: von Willebrand factor
Wnt9b: Wnt family member 9B
Zic3: Zic family member 3. Source: https://www.genecards.org retrieved on August 5 2020.
